# Structural basis of HCoV-19 fusion core and an effective inhibition peptide against virus entry

**DOI:** 10.1080/22221751.2020.1770631

**Published:** 2020-06-09

**Authors:** Huan Sun, Yan Li, Peipei Liu, Chengpeng Qiao, Xiaomin Wang, Lianao Wu, Kefang Liu, Yu Hu, Chao Su, Shuguang Tan, Shumei Zou, Guizhen Wu, Jinghua Yan, George Fu Gao, Jianxun Qi, Qihui Wang

**Affiliations:** a CAS Key Laboratory of Pathogenic Microbiology and Immunology, Institute of Microbiology, Chinese Academy of Sciences (CAS), Beijing, People’s Republic of China; b NHC Key Laboratory of Biosafety, National Institute for Viral Disease Control and Prevention, Chinese Center for Disease Control and Prevention, Beijing, People’s Republic of China; c CAS Key Laboratory of Microbial Physiological and Metabolic Engineering, Institute of Microbiology, Chinese Academy of Sciences, Beijing, People’s Republic of China; d University of Chinese Academy of Sciences, Beijing, People’s Republic of China; e Institute of Physical Science and Information, Anhui University, Hefei, People’s Republic of China; f College of Veterinary Medicine, China Agricultural University, Beijing, People’s Republic of China; g Savaid Medical School, University of Chinese Academy of Sciences, Beijing, People’s Republic of China

Dear editor,

Emerging and re-emerging pathogens are global challenges for public health [1]. Since the end of 2019, a cluster of coronavirus disease 2019 (COVID-19) cases have been reported, with a novel coronavirus (CoV), HCoV-19 as the causative agent [2]. Under exceptional control measures by the Chinese government, only a few new local cases have been confirmed recently. However, the virus has swiftly become a global pandemic and caused >234,000 confirmed cases, including >9800-related deaths globally as of 20 March 2020 (https://www.who.int/). Currently, there are no vaccines or therapeutics available.

Based on the knowledge on CoVs, the infection of HCoV-19 is mediated by first receptor recognition and then membrane fusion [3]. The spike (S) protein, which is likely to be cleaved into S1 and S2, is responsible for the two processes. S1 directly interacts with the receptor. The fusion is mediated by a 6-helix bundle fusion core constituted by the characteristic element called heptad repeats (HRs), HR1 and HR2 in S2. Peptides derived from HRs of SARS-CoV and MERS-CoV have been proven to be capable of inhibiting virus fusion and thereafter preventing virus infection [4,5]. In this study, we solved the crystal structure of fusion core of HCoV-19, and screened a panel of peptides that may potently inhibit virus infection.

We first positioned the HR sequences of HCoV-19 to residues E918-L966 for the HR1 region and residues D1163-L1203 for the HR2 region, using the SARS-CoV and MERS-CoV sequences as references [4,6] (Figure 1(a)). Recombinant proteins of fusion core, which consists HR1 and HR2 linked with a peptide linker, were expressed in *E. coli* cells and set up for crystal screening (Figure S1).

**Figure 1.**
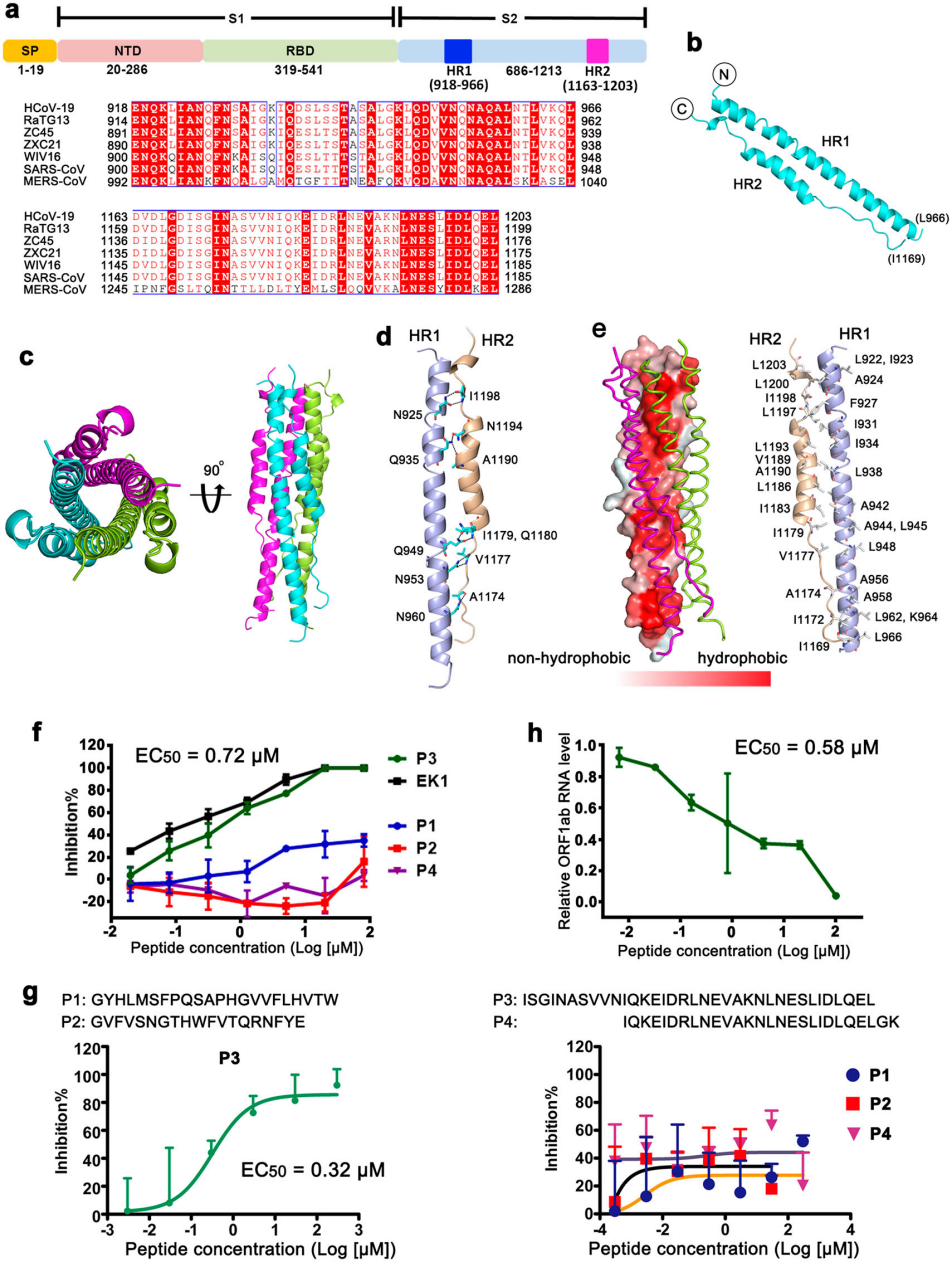
Structure of fusion core of HCoV-19 and identification of an inhibitory peptide for virus fusion. (a) Schematic representation of the extracellular region of HCoV-19 spike protein (S). SP, signal peptide. NTD, N-terminal domain. RBD, receptor-binding domain. Sequence alignment between HCoV-19, RaTG13, ZC45, ZXC21, WIV16, SARS-CoV and MERS-CoV S proteins for the HR1 and HR2 regions is shown. (b) Overall structure of the HR1/HR2 complex. (c) Six-helix bundle fusion core structure yielded by symmetry operations. The three HR1/HR2 chains are colored lemon, cyan, and magenta, respectively. Structures in top view (left panel) and side view (right panel) are presented, respectively. (d) The detailed interactions between HR1 and HR2. Residues involved in the hydrogen bond interactions were shown as sticks and labeled. (e) Hydrophobicity map was presented on the left panel. The two contacted HR1/HR2 complexes were illustrated with ribbon representation. The right showed the hydrophobic residues constituting this hydrophobic surface. (f) Inhibitory activity of P1, P2, P3 and P4 peptides, which were derived from S protein, against HCoV-19 S mediated cell–cell fusion. EK1 peptide as the positive control. Experiments were performed twice, and the data are expressed as means ± SD (error bar) of one representative result. (g) Identification of inhibitory peptides derived from S protein with pseudotyped viruses. The percent inhibition (*y*-axis) was plotted against the log value of peptide concentration (*x*-axis), and the fusion inhibition curves are shown. Each point represented the mean + SD from triplicate experiments. The EC_50_ values were listed. These results were representative data from two independent experiments. (h) Evaluation of P3 against live HCoV-19 infection. The relative ORF1ab RNA level of HCoV-19 (*y*-axis) was plotted against the log value of peptide concentration (*x*-axis). The amount of HCoV-19 in the samples was determined by RT-PCR. Values were means + SD of three samples per group. Experiments were performed twice and one representative data was displayed.

The structure of fusion core of HCoV-19 was determined at a resolution of 1.5 Å (Figure 1(b) and Table S1). The HR1 folds into a long α-helix while HR2 exhibits an extended structure from I1169 to Q1180 and folds into a canonical α-helical structure for the rest part. HR2 interacts with HR1 in an antiparallel manner. Overall, the structure of HR1 and HR2 complex of HCoV-19 resembles those of SARS-CoV and MERS-CoV (Figure S2), with root mean square deviation (RMSD) of ∼0.918 Å for 73 Cα atoms (superimposed with SARS-CoV) and ∼0.881 Å for 70 Cα atoms (superimposed with MERS-CoV). A trimeric structure of typical coiled-coil could be observed by simple symmetry operations with each HR1/HR2 complex in a crystallographic asymmetric unit (Figure 1(c)).

Detailed analysis of the interaction between HR1 and HR2 revealed that multiple hydrogen bond interactions were formed between amino acids from HR1 (N925, Q935, Q949, N953, N960) and amino acids from HR2 (A1174, V1177, I1179, Q1180, A1190, N1194, I1198) (Figure 1(d)). Previous studies have shown that the formation of hairpin trimer was mediated by the strong hydrophobic interface among the three HR complex units [4,5]. The HR motifs consist of a group of tandemly arranged seven-residue repeats and the repeatedly presented hydrophobic amino acids form a strong hydrophobic face to mediate HR hairpin trimerization. Hydrophobicity surface map of HCoV-19 HR1/HR2 complex was generated with a hydrophobic to non-hydrophobic color gradient based on classification of amino acid hydrophobicity properties (Figure 1(e)). The results indicate that an obvious hydrophobic face could be observed in the contacting face with the other two HR hairpins in the trimer. Further analysis of the atomic details revealed that multiple hydrophobic residues were presented in this hydrophobic face (Figure 1(e), right panel). On the other hand, no substantial hydrophobic regions could be observed in the other part of the surfaces free of HR hairpin interaction (Figure S3). Therefore, the formation of trimeric HR hairpin was mainly mediated by hydrophobic contacts involving residues from both HR1 and HR2.

It is believed that conformational rearrangement of HR regions occurs during the viral fusion process [3,7] and the peptides derived from the S2 have been shown to inhibit viral entry by interfering with the process of conformational transformation [4,5]. Thus, we designed four peptides, which were derived from S protein of HCoV-19 based on previous reports [4,8] and first evaluated their preventive effect from S protein-mediated cell–cell fusion [9]. As indicated in Figure 1(f), P3 peptide has exhibited substantial inhibitory effects with a 50% effective dose (EC_50_) of 0.72 μM. While the overlapped P4 peptide with P3 from HR2 and the other two peptides P1 and P2 located between HR1 and HR2 showed little inhibitory effect (Figure 1(f) and S4). We then tested their neutralizing activities against pseudotype HCoV-19 virus and the results revealed the EC_50_ of P3 is 0.32 μM (Figure 1(g)). Consistently, P3 also inhibited the authentic HCoV-19 virus infection to Vero E6 with the EC_50_ of 0.58 μM (Figure 1(h)). Thus, P3 inhibits HCoV-19 infection by preventing the S protein-mediated membrane fusion.

During the revision of this manuscript, a paper was published to report a structure of the fusion core of HCoV-19 S protein [10]. Superimposition of the two 6-helix bundle structures revealed an RMSD of 0.296 for 213 Cα atoms. Thus, the reported structural data supports the data in this manuscript and vice versa. The authors also reported a modified peptide, which is a potent pan-coronavirus fusion inhibitor targeting its S protein. Through rational design, they enhanced the activity of EK1 against HCoV-19 by shifting the EC_50_ from >2400 nM to 36.5 nM, which provides a good model for the modification of the P3 peptide. Considering the identical HR2 sequences between HCoV-19 and phylogenetically related bat CoVs, as well as SARS-CoV, P3 deserves further investigation and optimization as drug candidate to inhibit pan-SARS-CoV-like virus.

## Supplementary Material

Supplemental Material
