# Supplementary material for: Structural basis of HCoV-19 fusion core and an effective inhibition peptide against virus entry
Source: Emerg Microbes Infect. 2020 Jun 9;9(1):1238–41. doi: 10.1080/22221751.2020.1770631 (PMC7448930; doi:10.1080/22221751.2020.1770631)
Supplement: Supplemental Material [file TEMI_A_1770631_SM3345.docx]

Structural basis of HCoV-19 fusion core and an effective inhibition peptide against virus entry

Huan Sun^1,#^, Yan Li^1,#^, Peipei Liu^2,#^, Chengpeng Qiao^3^, Xiaomin Wang^1,4^, Lianao Wu^1,5^, Kefang Liu^1^, Yu Hu^1^, Chao Su^1^, Shuguang Tan^1^, Shumei Zou^2^, Guizhen Wu^2^, Jinghua Yan^1,3,6^, George Fu Gao^1^, Jianxun Qi^1,6^* and Qihui Wang^1,3^*

^1^ CAS Key Laboratory of Pathogenic Microbiology and Immunology, Institute of Microbiology, Chinese Academy of Sciences (CAS), Beijing 100101, China.

^2^ NHC Key Laboratory of Biosafety, National Institute for Viral Disease Control and Prevention, Chinese Center for Disease Control and Prevention, Beijing 102206, China

^3^ CAS Key Laboratory of Microbial Physiological and Metabolic Engineering, Institute of Microbiology, Chinese Academy of Sciences, Beijing 100101, China.

^4^ University of Chinese Academy of Sciences, Beijing 100049, China.

^5^ Institute of Physical Science and Information, Anhui University, Hefei 230039, China.

College of Veterinary Medicine, China Agricultural University, Beijing 100193, China.

^6^ Savaid Medical School, University of Chinese Academy of Sciences, Beijing 100049, China.

^#^ These authors contributed equally.

* Corresponding authors

**Supplementary information, Materials and Methods**

***Cell lines***

293T cells (for pseudotyped virus generation), human hepatoma cells (Huh7; for viral infection assay) and Vero E6 cells were cultured in Dulbecco’s modified Eagle’s medium (DMEM, Gibco) supplemented with 10% fetal bovine serum (FBS) (Gibco). All cell lines were maintained at 37°C in humidified air containing 5% CO_2_.

***Gene construction***

A construct with truncated HR1 and HR2 sequences were made involving HCoV-19 spike (GISAID No. EPI_ISL_402119 ) residues E918 to L966 for HR1 and D1163 to L1203 for HR2. The fusion core was constructed as a single chain by linking the HR1 and HR2 domains via a 22-amino-acid linker (LVPRGSGGSGGSGGLEVLFQGP). This flexible linker has been shown to work successfully in the SARS-CoV fusion core.^1^ The coding fragments were synthesized by Generay Biotech Co., Ltd., and inserted into the Nde I and Xho I restriction sites of the pET-21a vector (pET-21a-HCoV-19-HR1/HR2). The hexa-histidine tag coding sequence and the stop codon in the pET-21a vector were used for the construct.

The full-length coding sequence of HCoV-19 spike was cloned into the pCAGGS vector for pseudovirus production, named as pCAGGS-HCoV-19-S. HCoV-19 spike gene fused with eGFP at C terminal was cloned into pCDH vector for cell-cell fusion assay, named as pCDH-HCoV-19-S-eGFP. Two constructs were verified by direct DNA sequencing.

***Protein expression and purification***

For protein expression, pET-21a-HCoV-19-HR1/HR2 expression vectors were transformed into *Escherichia coli* strain BL21 (DE3) competent cells. A single colony was inoculated into 50 mL of Luria-Bertani (LB) medium containing 100 μg/mL of ampicillin and incubated overnight at 37°C. Then, the overnight culture was transferred to 2 liters of fresh LB medium for large-scale protein production by growing at 37°C. When the culture density (optical density at 600nm [OD600]) reached 0.6, protein over-expression was induced with 0.2 mM isopropyl-β-D-thiogalactoside (IPTG), and the cells were grown for an additional 10 h at 16°C before harvesting via centrifugation.

The collected bacterial cell pellet was resuspended in phosphate-buffered saline (PBS) and homogenized by sonication. The suspension was then centrifuged at 12,000 rpm for 30 min at 4°C. The supernatant was collected and then loaded onto a nickel-nitrilotriacetic acid (Ni-NTA) column (Beijing Zhongyuan L.T.D.). After removal of impurities by washing using PBS, the target protein was eluted with a buffer of 300 mM imidazole in PBS and then purified by gel filtration using a Superdex 200 10/300 GL column (GE) running on an Äkta Explorer fast-performance liquid chromatography (FPLC) system in a buffer composed of 20 mM Tris-HCl, pH 8.0, and 150 mM NaCl. The protein fractions were collected and analyzed on a 15% tricine SDS-PAGE gel. The molecular weights of the peak fractions were estimated by comparison with protein standards run on the same column.

***Crystallization, data collection, and structure determination***

The purified protein was concentrated to 5 mg/mL. Crystals of good diffracting quality were obtained after 3 days’ growth using the sitting drop vapor diffusion method by equilibrating a 2 μL drop (protein solution mixed 1:1 with reservoir solution) against a 100 μL reservoir containing 0.1 M bis-tris, pH 6.5, and 25% (wt/vol) polyethylene glycol 3350. Diffraction data were collected at Shanghai Synchrotron Radiation Facility (SSRF) BL17U1. For data collection, the crystals were cryo-protected by briefly soaking in reservoir solution supplemented with 20% (v/v) glycerol before flash-cooling in liquid nitrogen. The datasets were processed with HKL2000 software.^2^ The structure of the HCoV-19 HR1/HR2 complex was determined by molecular replacement with Phaser^3^ using the structure of the SARS-CoV fusion core (Protein Data Bank [PDB] code, 1WNC) as the search model. The initial atomic model was completed with Coot^4^ and refined with phenix.refine in Phenix,^5^ and the stereochemical qualities of the final model was assessed with MolProbity. Data collection, processing, and refinement statistics are summarized in Table S1. All structural figures were generated using Pymol software ([http://www.pymol.org](http://www.pymol.org/)).

***Pseudovirus neutralization assay***

HCoV-19 pseudovirus preparation and titration were performed as previously described^6^. Briefly, the plasmids of pCAGGS-HCoV-19-S and pNL4-3.luc.RE were co-transfected into HEK 293T cells cultured in 150 mm dish. After 48 h, the supernatant containing pseudovirus was harvested, centrifuged and stored at -80 °C. The 50% tissue culture infectious dose (TCID_50_) was determined by infection of Huh7 cells.

For the neutralization assay, 100 TCID_50_ /well pseudovirus was incubated with 10-fold serially diluted peptides (from 300 μM to 0.3 nM) for 30 min at 37 °C. The mixtures were then used to infect Huh7 cells seeded in 96-well plates with 3 repeats. After 4 h incubation, the FBS was added. Subsequently the medium was replaced with DMEM containing 10% FBS 2 h afterwards. The samples were incubated for an additional 40 h at 37 °C. Luciferase activity was measured using a GloMax 96 Microplate luminometer (Promega). The median effect concentration (EC_50_) was calculated using Prism (GraphPad).

***Inhibition of HCoV-19 S protein mediated cell-cell fusion.***

HCoV-19 S protein-mediated cell-cell fusion was assessed with a method as previously described^7^. Briefly, 293T cells were transfected with plasmid pCDH-HCoV-19-S-eGFP or pEGFP-N1 (negative control) and cultured in DMEM containing 10% FBS at 37 °C for 24 h. Cells were collected, and 3×10^4^ cells per 50 μL were incubated with the indicated peptides for 30 min. Then the mixture was added to the Huh-7 cells (5×10^4^) and incubated for another 2 h. 6 h later, photos of 3 independent fields were taken under the AMG EVOS FL digital inverted microscope and the fused and unfused cells were count, respectively. The concentration for 50% inhibition (IC50) was calculated using the GraphPad Prism6.0 software.

***Inhibition of live HCoV-19 replication***

The inhibition assay for live HCoV-19 was performed in a biosafety level 3 (BSL-3) facility at China Centers for Disease Control and Prevention. Peptides with diluted concentrations were mixed with 300×TCID_50_ HCoV-19 for 1 h and then added to the monolayer of Vero E6 cells with 3 replicates. After 48 h, 200 μL supernatants with indicated concentrations were collected and mixed with the lysis buffer for RNA isolation (TIANLONG Science and Technology Co., China). Quantitative RT-PCR was performed using HiScript II One Step qRT-PCRSYBR Green Kit (Vazyme, China) on an ABI Q5 (Applied Biosystems, US) according to the manufacturer’s protocol. Rnase P was used as the housekeeping gene to normalize samples. The analysis of relative levels of viral RNA in different samples was performed by comparative 2-ΔΔCT method.

**References:**

1. Xu Y. et al. *J. Biol. Chem.* **279**, 49414–49419 (2004).
2. Otwinowski Z, Minor W. *Methods Enzymol.* **276**, 307–326 (1997).
3. Read RJ. *Acta Crystallogr. D Biol. Crystallogr.* **57**, 1373–1382 (2001).
4. Emsley P, Cowtan K. *Acta Crystallogr. D Biol. Crystallogr.* **60**, 2126–2132 (2004).
5. Adams PD, *Acta Crystallogr. D* *Biol. Crystallogr.* **66**, 213–221 (2010).
6. Li, Y. et al. *Cell Res* **25**, 1237-1249 (2015).
7. Lu, L. et al. *Nat Commun* **5**, 3067. (2014).

Table S1. Data collection and refinement statistics

|  | Fusion core of the HCoV-19 |
| --- | --- |
| **Data collection** |  |
| Space group | P321 |
| Cell dimensions |  |
| *a*, *b*, *c* (Å) | 42.67, 42.67, 106.65 |
| α, β, γ (^o^) | 90.00, 90.00, 120.00 |
| Resolution (Å) | 50.00-1.50 (1.55-1.50) |
| Unique reflections  Completeness (%) | 18825 (1838)  100.0 (100.0) |
| *R*_merge_  *I* /σ*I* | 0.055 (0.452)  49.0 (7.1) |
| CC_1/2_ (%) | 1.000 (0.996) |
| Redundancy | 18.1 (13.8) |
|  |  |
| **Refinement** |  |
| Resolution (Å) | 21.62-1.50 |
| No. reflections | 17786 |
| *R*_work_ / *R*_free_ | 0.1912/0.2109 |
| No. atoms |  |
| Protein | 716 |
| Ligand/ion | 0 |
| Water | 112 |
| *B*-factors |  |
| Protein | 20.7 |
| Ligand/ion |  |
| Water | 36.5 |
| R.M.S. deviations |  |
| Bond lengths (Å) | 0.004 |
| Bond angles (^o^) | 0.605 |
| Ramchandran  Statistics (%)  Favored  Allowed  Disallowed | 98.85  1.15  0.00 |

Values in parentheses are for the highest resolution shell.

**Figure S1**

**
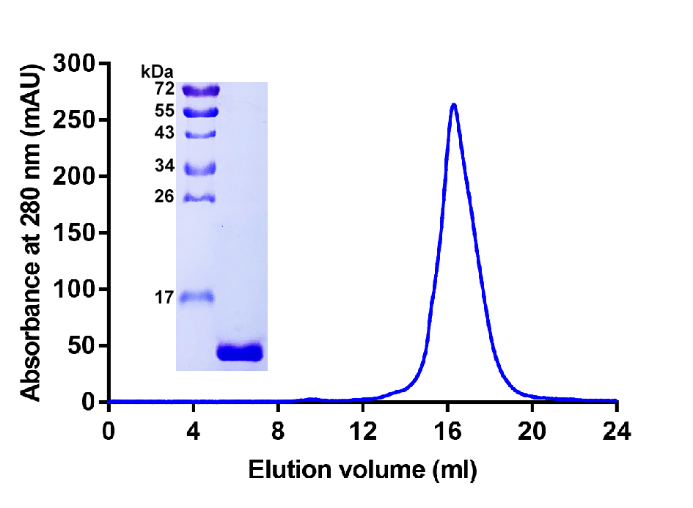
**

**Figure S1.** Gel filtration of HR1/HR21 complex and characterization by SDS-PAGE

**Figure S2**

**
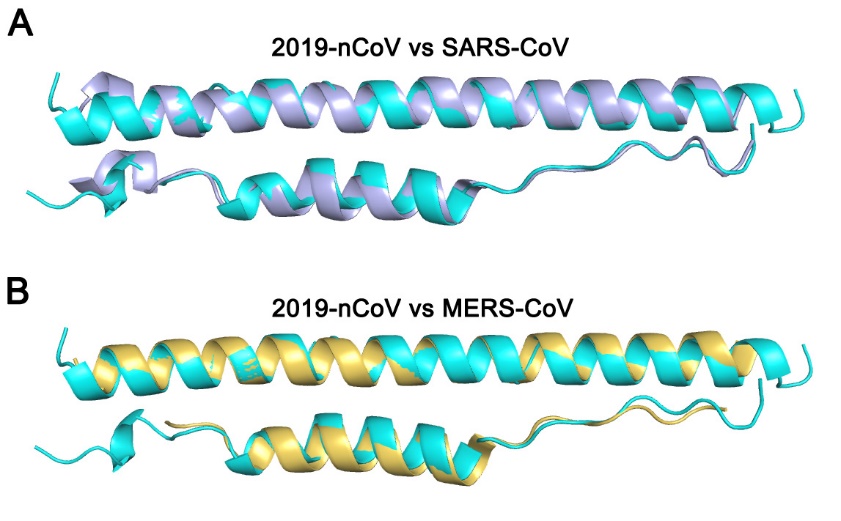
**

**Figure S2.** Structural comparisons of fusion cores. (A) Structural comparison between HCoV-19 and SARS-CoV fusion cores. (B) Structural comparison between HCoV-19 and MERS-CoV fusion cores. HCoV-19 is colored in cycan, SARS-CoV is colored in lightblue and MERS-CoV is colored in yellow.

**Figure S3**

**
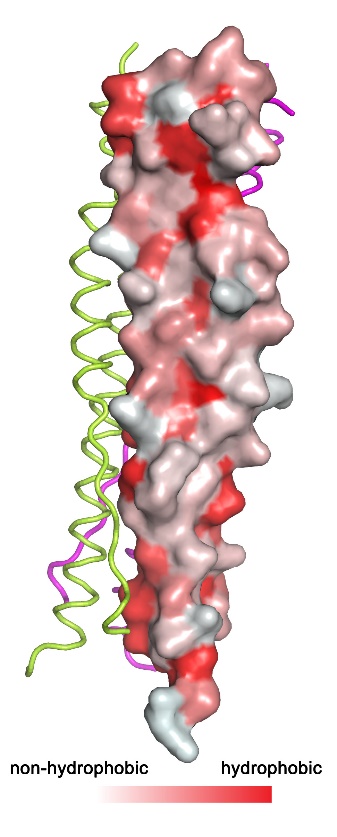
**

**Figure S3.** Hydrophobicity map of the surfaces free of HR hairpin interaction was shown with a hydrophobic to non-hydrophobic color gradient based on classification of amino acid hydrophobicity properties. The two contacted HR1/HR2 complexes were shown as ribbon.

**
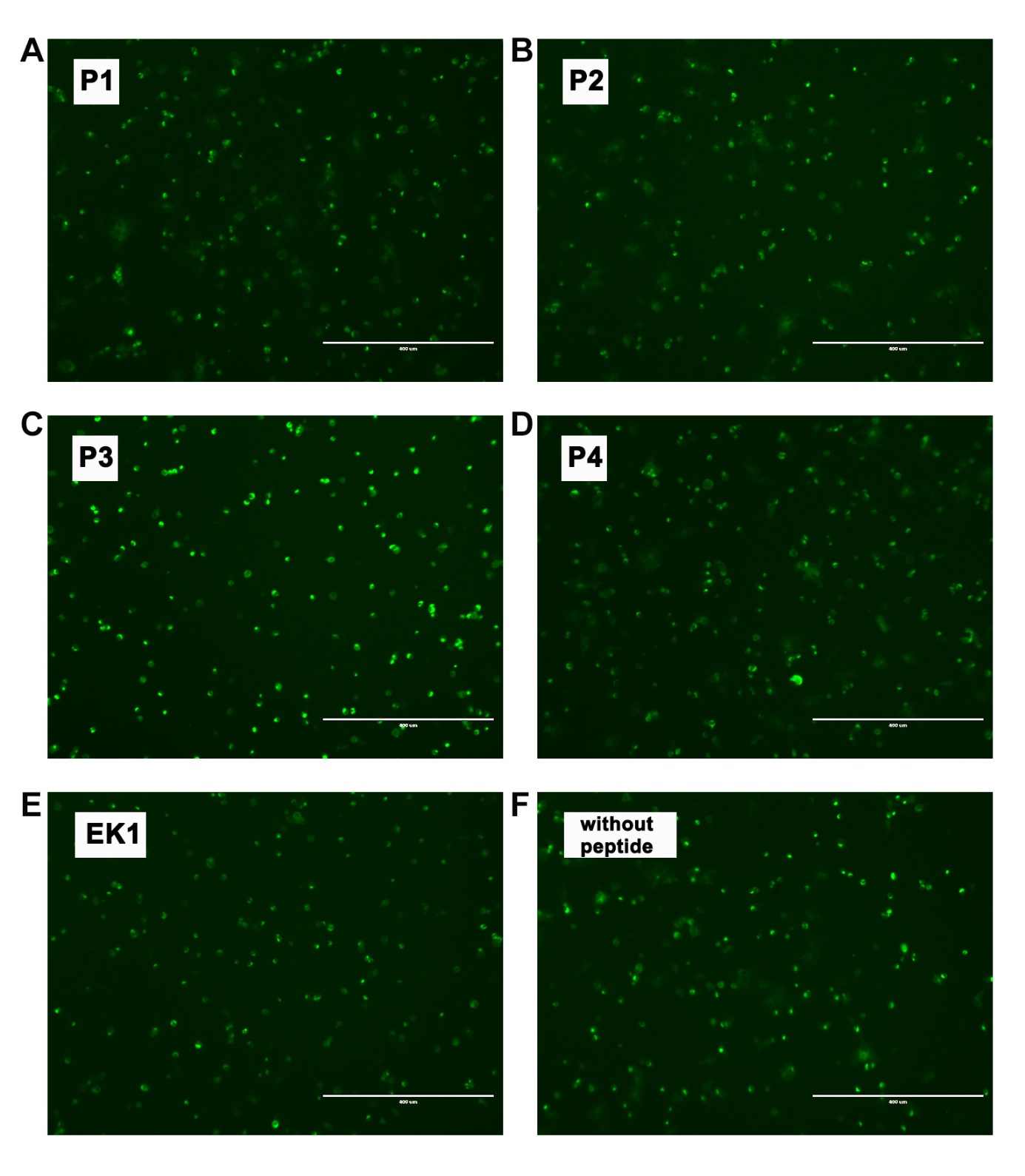
**

**Figure S4.** Inhibition of peptides against HCoV-19 S protein-mediated cell–cell fusion. Cell-cell fusion in the presence of P1 peptide (A), P2 peptide (B), P3 peptide (C), P4 peptide (D) and EK1 peptide (E) at 20 μM, and without peptide (F). (scale bar: 400 µm).
